# COVID-19: is fibrosis the killer?

**DOI:** 10.1007/s12079-020-00569-0

**Published:** 2020-05-13

**Authors:** Andrew Leask

**Affiliations:** School of Dentistry, University of Saskatchewan, 105 Wiggins Rd, Saskatoon, SK Canada

**Keywords:** COVID-19, Fibrosis, COPD, IL-6

## Abstract

COVID-19 is a respiratory disease. A recent report in Lancet examined, retrospectively, 137 patients with COVD-19. Patients that died had elevated IL-6 levels and acute respiratory distress syndrome. These data have obvious implications for how to control mortality in COVID-19.

Effective drugs that block and reverse lung fibrosis have not been identified. That COVID-19 is a respiratory disease is without question. A recent paper published in Lancet (Zhou et al. 2020) performed a retrospective study on patients at two hospitals in Wuhan, China. This paper was extremely data rich, but several points that were not adequately emphasized in this manuscript are of critical importance.

COVID-19 patients developed acute respiratory distress syndrome (ARDS), a disease that is characterized by rapid onset fibrosis. In this study, of 191 confirmed coronavirus patients, 50/54 patients that died had developed ARDS; conversely, only 9/137 survivors had ARDS. Moreover, the authors reported that chronic obstructive pulmonary disease (COPD) was cormorbid with COVID-19 (*p* = 0.056). Levels of interleukin (IL)-6, a proinflammatory cytokine linked with connective tissue disorders including fibrosis, were significantly higher in patients who died, and appeared to increase with disease progression. In survivors, essentially no alterations in IL-6 levels were observed.

These data strongly imply that not only is pulmonary fibrosis a comorbidity for COVID-19, but that it may be the cause of mortality for COVID-19. It also suggests that it may be reasonable to explore if IL-6 antagonists may be useful to treat COVID-19. In any event, these data strongly support the contention that development of anti-fibrotic agents is imperative not only to affect diseases such as scleroderma, idiopathic pulmonary fibrosis, Duchenne’s muscular dystrophy and metastatic cancers, but is also necessary to treat COVID-19.
